# Кандидатные SNP-маркеры, изменяющие сродство ТВР к промоторам Y-связанных генов CDY2A, SHOX, ZFY, снижают ряд показателей репродуктивного потенциала мужчин

**DOI:** 10.18699/VJ20.674

**Published:** 2020-11

**Authors:** M.P. Ponomarenko, E.B. Sharypova, I.A. Drachkova, L.K. Savinkova, I.V. Chadaeva, D.A. Rasskazov, P.M. Ponomarenko, L.V. Osadchuk, A.V. Osadchuk

**Affiliations:** Institute of Cytology and Genetics of Siberian Branch of the Russian Academy of Sciences, Novosibirsk, Russia Novosibirsk State University, Novosibirsk, Russia; Institute of Cytology and Genetics of Siberian Branch of the Russian Academy of Sciences, Novosibirsk, Russia; Institute of Cytology and Genetics of Siberian Branch of the Russian Academy of Sciences, Novosibirsk, Russia; Institute of Cytology and Genetics of Siberian Branch of the Russian Academy of Sciences, Novosibirsk, Russia; Institute of Cytology and Genetics of Siberian Branch of the Russian Academy of Sciences, Novosibirsk, Russia; Institute of Cytology and Genetics of Siberian Branch of the Russian Academy of Sciences, Novosibirsk, Russia; Institute of Cytology and Genetics of Siberian Branch of the Russian Academy of Sciences, Novosibirsk, Russia; Institute of Cytology and Genetics of Siberian Branch of the Russian Academy of Sciences, Novosibirsk, Russia; Institute of Cytology and Genetics of Siberian Branch of the Russian Academy of Sciences, Novosibirsk, Russia

**Keywords:** human, reproductive potential, chromosome Y, gene, promoter, TATA-binding protein (TBP), TBP-binding site(TATA-box), single nucleotide polymorphism (SNP), candidate SNP marker, verification *in vitro*, человек, репродуктивный потенциал, хромосома Y, ген, промотор, TATA-связывающий белок (TBP), TBP-связывающий сайт (ТATA-бокс), однонуклеотидный полиморфизм (SNP), SNP-маркер, верификация *in vitro*

## Abstract

Репродуктивный потенциал – уровень физического и психического состояния организма, позволяю- щий при достижении социальной зрелости воспроизводить здоровое потомство. В узком биомедицинском смыс- ле определение включает комплекс функциональных показателей репродуктивной системы, но в более широком смысле его можно рассматривать как совокупность физиологических, поведенческих, адаптивных, ментальных, ан- тропометрических и генетических характеристик особи, способствующих размножению. Целью настоящей работы было расширить область применимости созданного ранее Web-сервиса SNP_TATA_Z-tester для поиска кандидатных маркеров однонуклеотидного полиморфизма (SNP) на хромосоме Y человека, связанных с мужским репродуктив- ным потенциалом (МРП). В поиске кандидатных SNP-маркеров для МРП мы сосредоточились на генах хромосомы Y человека. Изучены 35 SNP в промоторах генов CDY2A, SHOX и ZFY, представляющих все три типа генов хромосомы Y человека: уникальный, псевдоаутосомный и паралог гена хромосомы X человека соответственно. Предсказаны 11 кандидатных SNP-маркеров ослабления МРП из-за изменения сродства TATA-связывающего белка (TBP) к этим промоторам. Выборочно верифицированы in vitro величины сродства «TBP-промотор», предсказанные в этой рабо- те. Установлена достоверная корреляция (r = 0.94, p < 0.005) между ними и результатами измерения in vitro сродства ТВР человека к олигонуклеотидам, идентичным сайтам ТВР-связывания исследуемых промоторов. Проведя поиск в базе данных PubMed по ключевым словам, мы нашли клиническое описание патологических состояний человека, соответствующих изменению экспрессии генов, несущих предсказанные нами кандидатные SNP-маркеры. Среди них оказались такие патологии, как нарушение сперматогенеза (ZFY: rs1388535808 и rs996955491), задержка поло- вого созревания (CDY2A: rs200670724), нарушения эмбриогенеза (SHOX: rs28378830) и непропорционально низкий рост с деформациями Маделунга (SHOX: rs1452787381). Они свидетельствуют, что в случае SNP-промоторов генов хромосомы Y человека следует ожидать изменений широкого круга показателей МРП, выходящих далеко за рамки генетического контроля собственно мужской репродуктивной функции.

## Abstract (en)

Reproductive potential is the most important conditional indicator reflecting the ability of individuals in a population to reproduce, survive and develop under optimal environmental conditions. As for humans, the concept of reproductive potential can include the level of the individual’s mental and physical state, which allows them to reproduce healthy offspring when they reach social and physical maturity. Female reproductive potential has been investigated in great detail, whereas the male reproductive potential (MRP) has not received the equal amount of attention as yet. Therefore, here we focused on the human Y chromosome and found candidate single-nucleotide polymorphism (SNP) markers of MRP. With our development named Web-service SNP_TATA_Z-tester, we examined in silico all 35 unannotated SNPs within 70-bp proximal promoters of the three Y-linked genes, CDY2A, SHOX and ZFY, which represent all types of human Y-chromosome genes, namely: unique, pseudo-autosomal, and human X-chromosome gene paralogs, respectively. As a result, we found 11 candidate SNP markers for MRP, which can significantly alter the TATA-binding protein (TBP) binding affinity for promoters of these genes. First of all, we selectively verified in vitro the values of the TBP-promoter affinity under this study, Pearson’s linear correlation between predicted and measured values of which were r = 0.94 (significance p < 0.005). Next, as a discussion, using keyword search tools of the PubMed database, we found clinically proven physiological markers of human pathologies, which correspond to a change in the expression of the genes carrying the candidate SNP markers predicted here. These were markers for spermatogenesis disorders (ZFY: rs1388535808 and rs996955491), for male maturation arrest (CDY2A: rs200670724) as well as for disproportionate short stature at Madelung deformity (e. g., SHOX: rs1452787381) and even for embryogenesis disorders (e. g., SHOX: rs28378830). This indicates a wide range of MRI indicators, alterations in which should be expected in the case of SNPs in the promoters of the human Y-chromosome genes and which can go far beyond changes in male fertility.

## Введение

Следуя идеям R.N. Chapman (1931) и E.R. Pianka (1976), популяционные экологи используют концепцию репро- дуктивного потенциала в качестве самого важного пока- зателя шансов воспроизвести потомков и довести их до репродуктивного возраста при наилучших условиях как на уровне индивида, так и на уровне популяции (Axelsson et al., 2010). Теория ожидаемой продолжительности жизни (Bowles, 1998) связывает репродуктивный потенциал, качество и продолжительность жизни человека с предрас- положенностью к болезням и стрессам, закодированной в его геноме. Наконец, прогресс медицины, достижения науки, развитие технологий и улучшение образования позволяют увеличить репродуктивный потенциал супер- популяции людей, хотя рост народонаселения, урбаниза- ция, загрязнение окружающей среды и эпидемии могут его уменьшить.

Базовая концепция предиктивно-превентивной пер- сонализированной медицины (Trovato, 2014), предполагающая достоверные различия между больными и здоровыми добровольцами по встречаемости у них клиниче-ских маркеров однонуклеотидного полиморфизма (SNP) (Varzari et al., 2016), указывает на возможность по рас- шифрованным индивидуальным геномам людей улуч- шить качество и продолжительность жизни как их, так и потомков. По SNP-маркерам генов, контролирующих показатели репродуктивного потенциала в геноме паци- ента, врач может прогнозировать способность к зачатию, предрасположенность к репродуктивным нарушениям, стрессорную реактивность и хронические болезни, спо- собные ухудшить его репродуктивное здоровье, качество жизни и долголетие, а также рекомендовать адекватный образ жизни, профилактические мероприятия, диету и форму взаимодействия пациента с врачом во избежание развития нежелательного жизненного сценария.

Крупнейший научный проект XXI в., «1000 геномов» (Telenti et al., 2016), как основа предиктивно-превентив- ной персонализированной медицины, уже выявил сотни миллионов SNP, собранных в базе данных dbSNP (Sherry et al., 2001), между десятками тысяч расшифрованных инди- видуальных геномов людей и эталонным (референсным) геномом человека в базе данных Ensembl (Cunningham et al., 2019). Наконец, база данных dbWGFP (Wu et al., 2016) собирает, систематизирует и приоритизирует сведения о каждом из всех 10 млрд потенциально возможных SNP человека в качестве первоосновы анализа индивидуального генома пациента в предиктивно-превентивной пер- сонализированной медицине (Trovato, 2014).

Поскольку врачебное решение на основе наличия или отсутствия тех или иных SNP-маркеров в геноме пациента нацелено на его здоровье, продолжительность и качество жизни, то приемлемы только биомедицинские SNP-маркеры, клинически доказанные сравнением больных и условно доровых людей (Varzari et al., 2016). Учет необходимых затрат времени, ручного труда и финансов для каждого такого теста исключает возможность оценить на практике проявление каждого из 10 млрд SNP человека (Wu et al., 2016) в патогенезе каждой из 55 тыс. болезней человека, согласно Международной классификации бо- лезней и сопутствующих проблем со здоровьем людей, МКБ- 11 (Pocai, 2019). Однако остается неясным, необходимо ли клинически проверять все SNP человека при условии, что дилемма J.B.S. Haldane (1957) и теория нейтральной эволюции (Kimura, 1968) предсказывают ней- тральность абсолютного большинства SNP человека. Сей- час для будущего клинического теста чаще всего вручную эвристически выбирают кандидатный SNP-маркер задан- ной патологии среди всех SNP генов, уже связанных с этой болезнью (Varzari et al., 2016). Выбор таких кандидатных SNP-маркеров можно сделать объективнее, быстрее и прицельнее за счет малозатратного биоинформатического выявления абсолютного большинства нейтральных SNP во всем референсном геноме человека (Ponomarenko M. et al., 2017). Хотя точность биоинформатических прогнозов для SNP все еще остается ниже порога их применимости в клинической практике (Yoo et al., 2015), она непрерывно увеличивается с каждым годом (Putlyaeva et al., 2018).

Сейчас наилучшую точность биоинформатических прогнозов достигли для SNP белок-кодирующих частей генов (Amberger et al., 2015), чье негативное влияние неустранимо ни сменой образа жизни, ни терапией (Mitsuyasu et al., 1998). В то же время значительно меньше исследованы SNP регуляторных районов генов (Zerbino et al., 2015), лишь модулирующих уровень экспрессии, что корректируемо как сменой образа жизни, так и лекарства- ми (Ponomarenko M. et al., 2013)). Поэтому регуляторные SNP сайта связывания ТАТА-связывающего белка (ТВР, каноническая форма сайта – ТАТА-бокс, ≈15 % ТАТА-со- держащих генов у человека), который необходим перед стартом любого транскрипта (Martianov et al., 2002; Rhee, Pugh, 2012) и который модулирует транскрипцию пропор- ционально сродству ТВР-промотор (Mogno et al., 2010), выглядят многообещающими в силу их предсказуемости и пользы для медицины (Ponomarenko M. et al., 2013, 2017).

Ранее мы создали Web-сервисы SNP_TATA_Comparator (Ponomarenko M. et al., 2015) и SNP_TATA_Z-tester (Sharypova et al., 2018) для оценки статистической значи- мости влияния SNP на сродство ТВР к промоторам генов человека и других эукариот с использованием библиотеки BioPerl (Stajich et al., 2002), доступа к базе данных Ensembl (Cunningham et al., 2019) и с помощью других источников данных соответственно. На этой методической основе мы предсказали кандидатные SNP-маркеры ожирения (Arkova et al., 2015), агрессивности (Chadaeva et al., 2016), нарушений циркадного ритма (Ponomarenko P. et al., 2016), атеросклероза (Ponomarenko M. et al., 2019), аутоиммун- ных заболеваний (Ponomarenko M. et al., 2016), болезни Альцгеймера (Ponomarenko P. et al., 2017), резистентности к противоопухолевой химиотерапии (Turnaev et al., 2016), предрасположенности к социальному доминированию и подчинению у людей (Chadaeva et al., 2019).

Целью настоящей работы было расширить область применимости наших Web-сервисов (Ponomarenko M. et al., 2015; Sharypova et al., 2018) для оценки SNP сайтов ТВР- связывания посредством поиска кандидатных SNP-маркеров на хромосоме Y человека, связанных с показателями мужского репродуктивного потенциала (МРП). Эта методология была использована при изучении женского репродуктивного потенциала (Chadaeva et al., 2018), тогда как МРП еще не получил должного внимания.

## Материалы и методы

**Биоинформатический анализ данных.** Мы исследо- вали последовательности ДНК S = {s_–70_…s_i_…s_–1_} дли- ной 70 п. о. перед стартами транскрипции (TSS ≡ s_0_; где s_i_∈{a, c, g, t}) промоторов белок-кодирующих генов хромосомы Y человека, как показано на рис. 1 и описано ранее, например в недавней работе (Ponomarenko M. et al., 2019). Стрелками на рис. 1 указано, как с помощью (а) Web-сервиса UCSC Genome Browser (Haeussler et al., 2015) и (б) базы данных dbSNP (Sherry et al., 2001) мы вводили исходные данные в наш (в) Web-сервис SNP_ TATA_Z-tester (Sharypova et al., 2018), где окно “Result” содержит результат обработки этих исходных данных в рамках нашей биоинформатической модели трехшагового связывания ТВР/промотор (Ponomarenko P. et al., 2008).

**Fig. 1.**
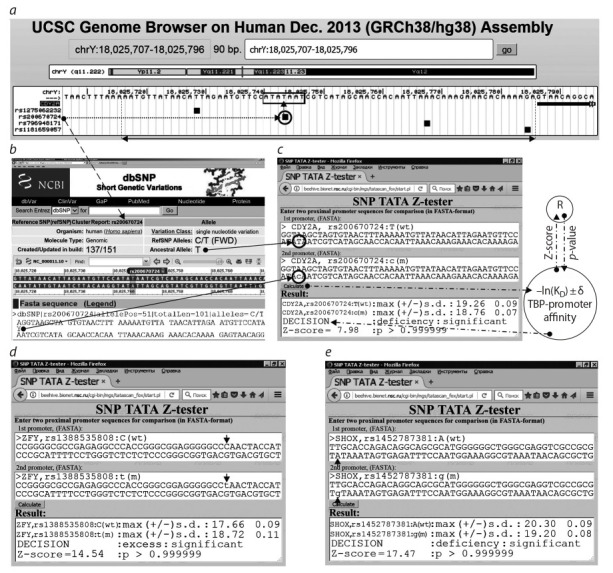
Analysis of the 70 base long proximal promoter (↔) upstream of the transcription start of the human CDY2A gene with four unannotated SNPs (■). One of them (rs200670724; dashed arrow) has been proposed as a candidate marker for human male maturation delay and poorer reproduction potential caused by poorer expression of the gene (Stahl et al., 2012) associated with the presence of the SNP in the TBP binding site (boxed). (a) Presentation of the human CDY2A promoter with the UCSC Genome Browser (Haeussler et al., 2015). (b) Annotation of rs200670724 SNP in dbSNP (Sherry et al., 2001). (c) The SNP_TATA_Z-tester application formerly developed by us. Line ”DECISION“ in its ”Result“ window present the prediction of CDY2A deficiency with the minor allele of the promoter as compared to the ancestral SNP variant, ® means the R software (Waardenberg et al., 2015). (d, e) Corresponding predictions of lower male reproductive potentials done by SNP_TATA_Z-tester for candidate SNP markers rs1388535808 and rs1452787381 in the ZFY and SHO genes, respectively.

Каждый SNP анализировали независимо от остальных таким образом, что если было предсказано недостоверное изменение сродства ТВР к минорному варианту промотора с этим SNP в сравнении с нормой, то он исключался из дальнейшего анализа (данные не показаны). Для оставшихся SNP с помощью стандартного поиска в базе данных PubMed (Lu, 2011) по ключевым словам нахо- дили сообщения о клинически значимых показателях МРП, которые соответствовали значимым изменениям экспрессии генов, несущих предсказанные кандидатные SNP, вследствие достоверного изменения сродства ТВР к промоторам этих генов.

**Эксперимент *in vitro.*** Рекомбинантный ТВР челове- ка (hTBP) экспрессировали в клетках Escherichia coli BL21(DE3) с плазмиды pAR3038-hTBP (любезно предо- ставлена проф. B. Puhg, The Pennsylvania State University, Pennsilvania, США) согласно (Peterson et al., 1990; Pugh, 1995), за исключением концентрации ИПТГ (1 мМ вместо 0.1 мМ) и времени индукции (3 ч вместо 1.5 ч).

В работе использовали олигодезоксирибонуклеотиды (ODN) длиной 26 п. о., синтезированные и дополнительно очищенные электрофорезом в ПААГ («Биоссет», Новоси- бирск). Для получения меченых двуцепочечных ODN обе цепи метили ^32^P-ATP («Биосан», Новосибирск) с помощью Т4-полинуклеотидкиназы («СибЭнзим», Новосибирск), отжигали при 95 °C (в эквимолярном соотношении) и медленно (не менее 3 ч) охлаждали до комнатной темпе- ратуры. Отожженные дуплексы очищали и анализировали электрофорезом в 15 % ПААГ в неденатурирующих усло- виях с последующей радиоавтографией на фосфоримиджере Molecular Imager PharosFX Plus (Bio-Rad) (Drachkova et al., 2005). Немеченые дуплексы получали так же и использовали без дополнительной очистки в ПААГ.

Для определения равновесной константы диссоциации (K_D_), а также времени полураспада (t_1/2_) констант ско- рости образования (k_a_) и распада (k_d_ ) комплексов ТВР- ODN проводили не менее трех экспериментов по связы- ванию ТВ-ODN при 25 °C в буфере, содержащем 20 мМ HEPES-KOH (pH 7.6), 5 мМ MgCl_2_, 70 мМ KCl, 1 мМ DTT, 100 мкг/мл BSA, 0.01 % NP-40, и 5 % глицерина с фиксированной концентрацией 2 нМ активного TBP. Каждый эксперимент включал 32 реакции связывания (8 временных точек, каждая с четырьмя концентрациями ODN). Четверка реакций связывания (1 временная точка) запускалась одновременно добавлением TBP к реакционной смеси, содержащей ODN, и немедленным переносом в термостат на 25 °C. По окончании реакции связывания все реакционные смеси наносили на ПААГ одновременно при напряженности поля 10 В/см. Комплек- сы ТВР-ODN отделяли от несвязанного ODN с помощью количественного метода электрофоретической задержки (EMSA). Электрофорез проводили в нативном 5 % ПААГ на Tris-глициновом буфере (рН 8.3) в течение 40 мин при температуре 10 °C и напряженности поля 25 В/см.

Затем гели высушивали и экспонировали с экраном Imaging Screen-K (Kodak) для фосфоимиджера Molecular Imager PharosFX Plus (Bio-Rad). Экран сканировали на фосфоимиджере и с помощью программы Quantity One – 4.5.0 (Bio-Rad) проводили количественный анализ радиоавтографов. Полученные зависимости концентрации комплексов ТВР-ODN от концентраций ODN вводили как исходные данные в общедоступный Web-сервис GraphPad Prism 5 (http://graphpad-prism.software.informer.com/5.01), результатом которого были величины K_D_, t_1/2_, k_a_ и k_d_ анализируемых комплексов ТВР-ODN, а также оценки стандартных ошибок средних (± SEM) для этих величин.

**Статистический анализ.** Биоинформатические оценки сродства TBP к вариантам SNP-промоторов генов хро- мосомы Y человека сравнивали с экспериментальными величинами сродства ТВР к ODN, идентичным этим про- моторам, с помощью STATISTICA (Statsoft™).

## Результаты и обсуждение

Проанализированы все 35 неаннотированных SNP, до- ступных в выпуске № 151 базы данных dbSNP (Sherry et al., 2001), для проксимальных промоторов длиной 70 п. о. генов CDY2A, SHOX и ZFY, представляющих все три типа генов на хромосоме Y человека: уникальный, псевдоаутосомный и паралог гена хромосомы X человека соответственно (табл. 1). В результате было выявлено 11 кандидатных SNP-маркеров снижения МРП из-за до- стоверных изменений сродства ТВР к этим промоторам (см. табл. 1). Рассмотрим сначала наиболее подробно единственный кандидатный SNP-маркер rs200670724 в гене CDY2A, связанный с ослаблением МРП, чтобы затем, благодаря аналогиям с этим иллюстративным примером, кратко описать 10 остальных кандидатных SNP-маркеров в генах ZFY и SHOX.

**Table 1.**
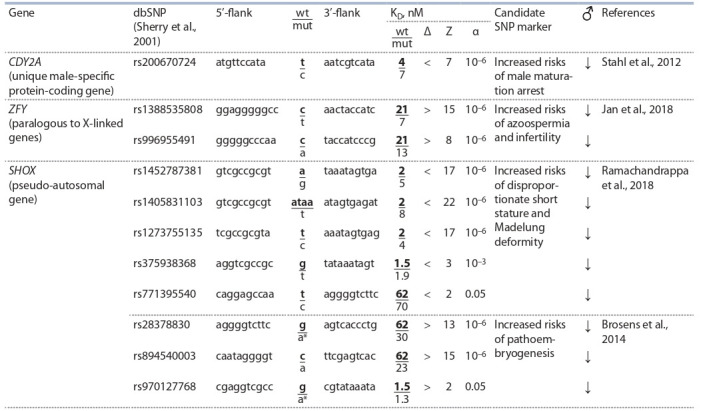
Candidate SNP markers of male reproductive potential (MRP) capable of altering the affinity of TBP for the promoters of the human Y chromosome genes (according to our predictions made in this work) Notе. wt – norm (reference human genome); mut – minor allele; K_D_ – equilibrium dissociation constant of TBP-promoter complex; Δ – alteration in the affinity of TBP for promoter, namely: increase (<) and decrease (>); Z – Z-value of Fisher’s Z-test; α – statistical significance estimate (α = 1 – p, where: p, probability estimate for random reasons shown in Fig. 1, d ); ♂ – alteration in MRP, namely: increase (↑) and decrease (↓). Genes: CDY2A – chromodomain Y-linked 2A; ZFY – zinc finger protein Y-linked; SHOX – short stature homeobox. * In addition to the indicated minor allele, other alleles are present, which insignificantly alter the affinity of TBP to the corresponding human promoter.

Уникальный **ген CDY2A** на хромосоме Y человека ко- дирует хромодомен 2А и несет четыре неаннотированных SNP в своем проксимальном промоторе длиной 70 п. о. (см. рис. 1, а), лишь один среди которых, rs2276109 (см. рис. 1, б ), достоверно снижает экспрессию этого гена, со- гласно нашему прогнозу (см. рис. 1, в). Как представлено в первой строке табл. 1, этот дефицит экспрессии гена CDY2A вызван снижением сродства «ТВР-промотор» с 4 нМ в норме до 7 нМ для минорного аллеля rs2276109.

Прежде всего мы экспериментально проверили этот прогноз методом задержки электрофоретической подвиж- ности (EMSA). Электрофореграммам для нормального и минорного аллелей rs2276109 соответствуют рис. 2, a и рис. 2, б, тогда как зависимость концентрации комплексов ТВР-ODN от концентраций ODN, которые были построены на основе этих электрофореграмм, демонстрируют рис. 2, в и 2, г.

На этих рисунках можно видеть снижение сродства “ТВР-ODN” с 160 нМ в норме до 500 нМ для минорного аллеля rs2276109. Это свидетельствует об адекватности оценок Web-сервиса SNP_TATA_Z-tester (Sharypova et al., 2018) для оценки сродства ТВР к промоторам генов на хромосоме Y человека.

Наконец, с помощью поиска по ключевым словам в базе данных PubMed (Lu, 2011) мы нашли клинические данные (Stahl et al., 2012) о дефиците экспрессии гена CDY2A при задержке полового созревания. Это позволило нам предложить кандидатный SNP-маркер rs200670724 как генетическую причину снижения МРП вследствие задержки полового созревания мужского организма.

**Ген ZFY** (Y-связанный белок «цинковый палeц») яв- ляется паралогом гена ZFX на хромосоме X человека. В его промоторе мы нашли два неаннотированных SNP, rs1388535808 и rs996955491, способных вызвать избыток ZFY, как представлено на рис. 1, г.

S.Z. Jan с коллегами (2018) сообщили о суперэкспрес- сии ZFY в сперматоцитах как клиническом маркере оста- новки их мейоза, азооспермии и бесплодия у мужчин. В границах применимости этих клинических наблюдений без гипотез о причинно-следственных связях мы предска- зали rs1388535808 и rs996955491 как кандидатные SNP- маркеры, повышающие риск остановки сперматогенеза на стадии мейоза с последующей азооспермией и бес- плодием, что безусловно, значительно снижает мужской репродуктивный потенциал (см. табл. 1).

**Ген SHOX** (гомеобокс низкорослости) локализован в псевдо-аутосомном районе 1 (PAR1) хромосомы Y чело- века. В проксимальных промоторах длиной 70 п. о. перед его стартами транскрипции мы нашли пять кандидатных SNP-маркеров для дефицита этого транскрипционного фактора (см. рис. 1, e: rs1452787381) и три кандидатных SNP-маркера для его избытка (см. табл. 1: rs28378830). Согласно табл. 1, они снижают МРП посредством непро- порционально низкого роста с деформациями Маделунга (Ramachandrappa et al., 2018) либо нарушений эмбриоге- неза (Brosens et al., 2014) соответственно.

Экспериментальные данные *in vitro* для выборочной проверки трех кандидатных SNP-маркеров сниже- ния МРП, rs20067072 (CDY2A), rs1452787381 (ZFY ) и rs1452787381 (SHOX ), представлены в табл. 2. С исполь- зованием метода EMSA были измерены, прежде всего, равновесная константа диссоциации (K_D_), а также время полураспада (t_1/2_), константы скорости сборки (k_a_) и рас- пада (k_d_ ) комплексов ТВР-ODN, как это было показано на иллюстративном примере rs200670724 (см. рис. 2).

**Fig. 2.**
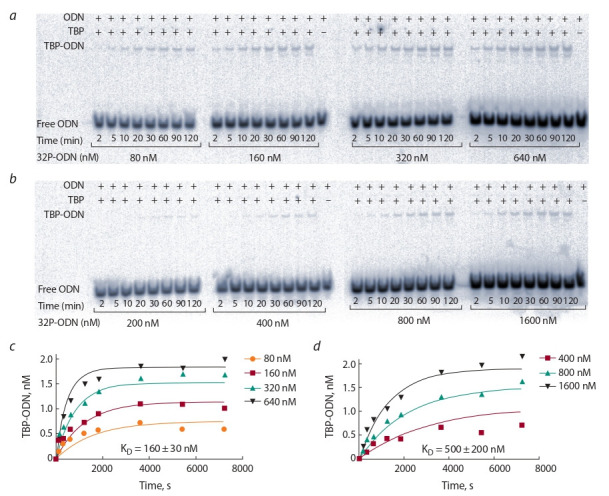
Measuring the kinetics of TBP binding to two TATA-containing ODNs identical to the human CDY2A gene promoter. Legend: (a and b) Electropherograms of the wild-typed ancestral and minor alleles of the unannotated SNP rs200670724 under this study, respectively; the concentration of TBP was 2 nM in all the experiments; the concentrations of an ODN containing a tested SNP allele that we used are indicated; (c и d ) dependences of reaction rates on ODN concentrations in the cases of the ancestral and minor alleles of the SNP rs200670724, respectively; the K_D_ value of the equilibrium dissociation constant was inferred from the dependences of reaction rates on ODN concentrations according to the Web-service GraphPad Prism 5 (http://graphpad-prism.software.informer.com/5.01).

Достоверные робастные корреляции – линейная Пир- сона (r), обобщенная Гудмана–Крускала (γ), ранговые Спирмена (R) и Кендалла (τ), – между нашими предска- занными (см. табл. 1) и экспериментальными (см. табл. 2) значениями равновесной константы диссоциации (K_D_), выраженными в натуральных логарифмических единицах, показаны на рис. 3. Они доказывают адекватность наших прогнозов для величин сродства ТВР к промоторам генов Y-хромосомы человека. В порядке обсуждения можно коротко отметить, что несовпадение между шкалами вертикальной (эксперимент) и горизонтальной (прогноз) осей на рис. 3 соответствует различию в концентрациях TBP (т. е. в неконтролируемой доле димеров TBP, которые име- ют K_D_ = 4 ± 1.5 нМ (Colemanet al., 1995) и не связывают ДНК) в этой работе (2 нМ) и ранее (Ponomarenko Р. et al., 2008) при оптимизации трехшаговой модели связывания «ТВР-промотор» (0.3 нМ), использованной Web-сервисом SNP_TATA_Z-tester (Sharypova et al., 2018) для прогнозов в этой работе.

**Fig. 3.**
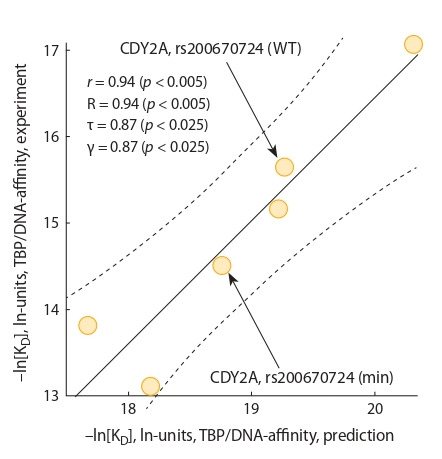
The significant robust correlations between the predicted in silico and experimentally measured in vitro values of the TBP–DNA affinity (in natural logarithmic units). Legend: solid and dashed lines denote the linear regression and boundaries of its 95 % confidence interval, calculated by means of standard software package STATISTICA (Statsoft™, Tulsa, USA); arrows pinpoint the ancestral (WT) and minor (min) alleles of the SNP being studied (rs200670724 of CDY2A ), an analysis of which is depicted in Figures 1 and 2 as an example of the application of our Web service SNP_TATA_Z-tester (Sharypova et al., 2018) in this work and its in vitro selective verification here; r, γ, R, τ , and p are coefficients of linear correlation, Goodman–Kruskal generalized correlation, Spearman’s and Kendall’s rank correlations, and their p values, respectively.

**Table 2.**
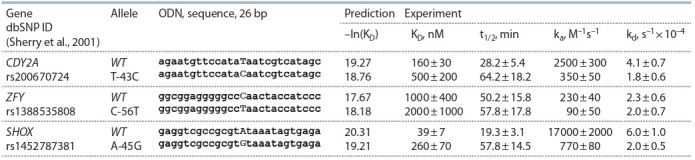
EMSA-based in vitro analysis of a complex of TBP and synthetic 26 bp ODNs identical to natural promoters near the SNPs being tested Note: t_1/2_, half-life; K_D_, dissociation constant; k_a_ and k_d_, assembly and dissociation rate constants, respectively.

Самая близкая к канонической последовательность ТАТА-бокса наблюдается в промоторе гена SHOX с K_D_ = 39 ± 7 нм (см. табл. 2). Минорный вариант SNP rs1452787381 (замена «A» на «G» в ТАТА-боксе) приводит к снижению сродства почти в 7 раз, что обусловлено сни- жением скорости образования комплексов (k_a_) в 22 раза. Скорость распада комплексов (k_d_) снижается в 3 раза, а также увеличивается время жизни комплексов (t_1/2_). Все эти характеристики могут привести к повышенному ри- ску формирования непропорционально низкого роста с деформациями Маделунга.

Наихудшее сродство “ТВР-OND”, K_D_ = 1000 ± 400 нм, было определено для комплексов ТВР с промотором гена ZFY, имеющего последовательность (СС**С**ААСТАС), лишь весьма отдаленно напоминающую консенсус ТАТА бокса (ТАТААААG). Замена «С» на «Т» не спасает поло- жение – скорость образования комплексов (k_a_) снижается в 2.5 раза, и незначительно изменяется скорость их рас- пада (k_d_ ) и время жизни (t_1/2_). Можно предположить, что предсказанная нами *in silico* суперэкспрессия минорного аллеля SNP rs1388535808 в сперматоцитах, приводящая к остановке сперматогенеза на стадии мейоза, азооспермии и бесплодию, в условиях *in vivo* обеспечивается участи- ем ТВР-ассоциированных факторов (TAFs) и других регуляторных белков в образовании транскрипционного комплекса в случае ТАТА-несодержащего промотора гена ZFY человека.

Полученные данные о снижении сродства ТВР к про- мотору гена CDY2A более чем в 3 раза для аллеля С соответствуют клиническим данным о дефиците продукта этого гена как генетической причине задержки полового созревания.

## Заключение

В настоящей работе исследованы все 35 SNP в промоторах генов CDY2A, SHOX и ZFY, представлявших все три типа генов хромосомы Y человека: уникальный, псевдоаутосомный и паралог гена хромосомы X человека соответственно. Из них отобраны 11 кандидатных SNP-маркеров, способных изменить сродство TBP к этим промоторам и таким образом способствовать ослаблению МРП. Прежде всего, мы выборочно верифицировали in vitro величины сродства «TBP-промотор», предсказанные в этой работе, и установили статистически достоверную линейную корреляцию между ними и экспериментально измеренными величинами (r = 0.94, p < 0.005). Используя поиск по ключевым словам в базе данных PubMed, мы нашли клинические данные о физиологических признаках патологий человека, которые соответствовали изменению экспрессии генов, несущих предсказанные кандидатные SNP-маркеры. Среди них были показатели процесса спер- матогенеза (ZFY: rs1388535808 и rs996955491), задержки полового созревания (CDY2A: rs200670724), непропор- ционально низкого роста с деформациями Маделунга (например, SHOX: rs1452787381) и нарушения процесса эмбриогенеза (например, SHOX: rs28378830). В совокупности полученные результаты свидетельствуют, что в случае SNP-промоторов генов хромосомы Y человека следует ожидать изменений широкого круга показателей МРП, выходящих далеко за рамки генетического контроля соб- ственно мужской репродуктивной функции.

Это заключение согласуется с выводом авторов рекон- струкции коэволюции хромосом X и Y у разных видов (опоссум, бык, крыса, мышь, мармозетка, макака-резус, шимпанзе и человек) на основе данных высокопроизводительного секвенирования (Bellott et al., 2014), что, кроме мужской репродуктивной функции, хромосома Y определяет также гендерный диморфизм в предрасположен- ности к заболеваниям и жизнеспособность мужчин. Предсказанные и экспериментально подтвержденные здесь изменения сродства ТВР к минорным вариантам SNP генов CDY2A, SHOX и ZFY человека могут снизить МРП лишь при аномальных изменениях уровней белков, ко- дируемых этими генами, в соответствующих тканях на определенных стадиях онтогенеза. Это с необходимостью требует клинической проверки, на снижение затрат кото- рой благодаря улучшению ее адресности нацелено наше экспериментально-биоинформатическое исследование.

## Conflict of interest

The authors declare no conflict of interest.

## References

Amberger J., Bocchini C., Schiettecatte F., Scott A., Hamosh A. OMIM.
org: Online Mendelian Inheritance in Man (OMIM®), an online
catalog
of human genes and genetic disorders. Nucleic Acids Res.
2015;43(D1):D789-D798. DOI 10.1093/nar/gku1205.

Arkova O.V., Ponomarenko M.P., Rasskazov D.A., Drachkova I.A., Arshinova
T.V., Ponomarenko P.M., Savinkova L.K., Kolchanov N.A.
Obesity-related known and candidate SNP markers can significantly
change affinity of TATA-binding protein for human gene promoters.
BMC Genomics. 2015;16(Suppl. 13):S5. DOI 10.1186/1471-2164-
16-S13-S5.

Axelsson J., Bonde J.P., Giwercman Y.L., Rylander L., Giwercman A.
Gene-environment interaction and male reproductive function.
Asian J. Androl. 2010;12(3):298-307. DOI 10.1038/aja.2010.16.

Bellott D.W., Hughes J.F., Skaletsky H., Brown L.G., Pyntikova T., Cho
T.-J., Koutseva N., Zaghlul S., Graves T., Rock S., Kremitzki C.,
Fulton R.S., Dugan S., Ding Y., Morton D., Khan Z., Lewis L., Buhay
C., Wang Q., Watt J., Holder M., Lee S., Nazareth L., Alfoldi J.,
Rozen S., Muzny D.M., Warren W.C., Gibbs R.A., Wilson R.K.,
Page D.C. Mammalian Y chromosomes retain widely expressed
dosage-sensitive regulators. Nature. 2014;508(7497):494-499. DOI
10.1038/nature13206.

Bowles J.T. The evolution of aging: a new approach to an old problem
of biology. Med. Hypotheses. 1998;51(3):179-221. DOI 10.1016/
s0306-9877(98)90079-2.

Brosens E., de Jong E., Barakat T., Eussen B., D’Haene B., De Baere
E., Verdin H., Poddighe P., Galjaard R., Gribnau J., Brooks A.,
Tibboel
D., de Klein A. Structural and numerical changes of chromosome
X in patients with esophageal atresia. Eur. J. Hum. Genet.
2014;22:1077-1084. DOI 10.1038/ejhg.2013.295.

Chadaeva I.V., Ponomarenko M.P., Rasskazov D.A., Sharypova E.B.,
Kashina E.V., Matveeva M.Yu., Arshinova T.V., Ponomarenko P.M.,
Arkova O.V., Bondar N.P., Savinkova L.K., Kolchanov N.A. Candidate
SNP markers of aggressiveness-related complications and comorbidities
of genetic diseases are predicted by a significant change
in the affinity of TATA-binding protein for human gene promoters.
BMC Genomics. 2016;17(Suppl. 14):995. DOI 10.1186/s12864-
016-3353-3.

Chadaeva I., Ponomarenko P., Rasskazov D., Sharypova E., Kashina
E., Zhechev D., Drachkova I., Arkova O., Savinkova L., Ponomarenko
M., Kolchanov N., Osadchuk L., Osadchuk A. Candidate
SNP markers of reproductive potential are predicted by a significant
change in the affinity of TATA-binding protein for human gene
promoters. BMC Genomics. 2018;19(Suppl. 3):19. DOI 10.1186/
s12864-018-4478-3.

Chadaeva I., Ponomarenko P., Rasskazov D., Sharypova E., Kashina E.,
Kleshchev M., Ponomarenko M., Naumenko V., Savinkova L., Kolchanov
N., Osadchuk L., Osadchuk A. Natural selection equally supports
the human tendencies in subordination and domination: a genome-
wide study with in silico confirmation and in vivo validation
in mice. Front. Genet. 2019;10:73. DOI 10.3389/fgene.2019.00073.
Chapman R.N. Animal Ecology with Special Reference to Insects.
N. Y.; London: McGraw-Hill Book Co Inc, 1931.

Coleman R., Taggart A., Benjamin L., Pugh B. Dimerization of the
TATA binding protein. J. Biol. Chem. 1995;270:13842-13849.
Cunningham F., Achuthan P., Akanni W., Allen J., Amode M.,
Armean I., Bennett R., Bhai J., Billis K., Boddu S., Cummins C.,
Davidson C., Dodiya K., Gall A., Giron C., Gil L., Grego T., Haggerty
L., Haskell E., Hourlier T., Izuogu O., Janacek S., Juettemann
T., Kay M., Laird M., Lavidas I., Liu Z., Loveland J., Marugan
J., Maurel T., McMahon A., Moore B., Morales J., Mudge J.,
Nuhn M., Ogeh D., Parker A., Parton A., Patricio M., Abdul
Salam A., Schmitt B., Schuilenburg H., Sheppard D., Sparrow H.,
Stapleton E., Szuba M., Taylor K., Threadgold G., Thormann A.,
Vullo A., Walts B., Winterbottom A., Zadissa A., Chakiachvili M.,
Frankish A., Hunt S., Kostadima M., Langridge N., Martin F.,
Muffato M., Perry E., Ruffier M., Staines D., Trevanion S., Aken B.,
Yates A., Zerbino D., Flicek P. Ensembl 2019. Nucleic Acids Res.
2019;47(D1):D745-D751. DOI 10.1093/nar/gky1113.

Drachkova I.A., Lysova M.V., Repkova M.N., Prokuda O.V., Sokolenko
A.A., Arshinova T.V., Kobzev V.F., Iamkovoĭ V.I., Savinkova
L.K. Interaction of proteins from general transcription complex
RNA polymerase II with oligoribonucleotides. Mol. Biol. (Mosk).
2005;39(1):139-146.

Haeussler M., Raney B.J., Hinrichs A.S., Clawson H., Zweig A.S., Karolchik
D., Casper J., Speir M.L., Haussler D., Kent W.J. Navigating
protected genomics data with UCSC Genome Browser in a box.
Bioinformatics. 2015;31(5):764-766. DOI 10.1093/bioinformatics/
btu712.

Haldane J.B.S. The cost of natural selection. J. Genet. 1957;55:511-
524. DOI 10.1007/BF02984069.

Jan S.Z., Jongejan A., Korver C.M., van Daalen S.K.M., van Pelt A.M.M.,
Repping S., Hamer G. Distinct prophase arrest mechanisms in human
male meiosis. Development. 2018;145(16):dev160614. DOI
10.1242/dev.160614.

Kimura M. Evolutionary rate at the molecular level. Nature. 1968;
217(5129):624-626. DOI 10.1038/217624a0.

Lu Z. PubMed and beyond: a survey of web tools for searching biomedical
literature. Database (Oxford). 2011;2011:baq036. DOI
10.1093/database/baq036.

Martianov I., Viville S., Davidson I. RNA polymerase II transcription
in murine cells lacking the TATA binding protein. Science. 2002;
298(5595):1036-1039. DOI 10.1126/science.1076327.

Mitsuyasu H., Izuhara K., Mao X., Gao P., Arinobu Y., Enomoto T.,
Kawai M., Sasaki S., Dake Y., Hamasaki N., Shirakawa T., Hopkin
J. Ile50Val variant of IL4R-alpha upregulates IgE synthesis and
associates with atopic asthma. Nat. Genet. 1998;19:119-120. DOI
10.1038/472.

Mogno I., Vallania F., Mitra R.D., Cohen B.A. TATA is a modular
component of synthetic promoters. Genome Res. 2010;20(10):1391-
1397. DOI 10.1101/gr.106732.110.

Peterson M.G., Tanese N., Pugh B.F., Tjian R. Functional domains and
upstream activation properties of cloned human TATA binding protein.
Science. 1990;248(4963):1625-1630.

Pianka E.R. Natural selection of optimal reproductive tactics. Am. Zool.
1976;16(4):775-784. www.jstor.org/stable/3882142.

Pocai B. The ICD-11 has been adopted by the World Health Assembly.
World Psychiatry. 2019;18(3):371-372. DOI 10.1002/wps.20689.

Ponomarenko M., Arkova O., Rasskazov D., Ponomarenko P., Savinkova
L., Kolchanov N. Candidate SNP markers of gender-biased
autoimmune complications of monogenic diseases are predicted by
a significant change in the affinity of TATA-binding protein for human
gene promoters. Front. Immunol. 2016;7:130. DOI 10.3389/
fimmu.2016.00130.

Ponomarenko M., Mironova V., Gunbin K., Savinkova L. Hogness
Box. In: Maloy S., Hughes K. (Eds.). Brenner’s Encyclopedia of Genetics.
2nd edn. San Diego: Academic Press, Elsevier Inc. 2013;3:
491-494. DOI 10.1016/B978-0-12-374984-0.00720-8.

Ponomarenko M., Rasskazov D., Arkova O., Ponomarenko P., Suslov
V., Savinkova L., Kolchanov N. How to use SNP_TATA_Comparator
to find a significant change in gene expression caused by the
regulatory SNP of this gene’s promoter via a change in affinity of
the TATA-binding protein for this promoter. Biomed. Res. Int. 2015;
2015:35983004625. DOI 10.1155/2015/359835.

Ponomarenko M., Rasskazov D., Chadaeva I., Sharypova E., Drachkova
I., Ponomarenko P., Oshchepkova E., Savinkova L., Kolchanov
N. Candidate SNP markers of atherosclerosis that may significantly
change the affinity of the TATA-Binding protein for the
human gene promoters. Russ. J. Genet. 2019;55(9):1137-1151. DOI
10.1134/s1022795419090114.

Ponomarenko M., Rasskazov D., Chadaeva I., Sharypova E., Ponomarenko
P., Arkova O., Kashina E., Ivanisenko N., Zhechev D., Savinkova
L., Kolchanov N. SNP_TATA_Comparator: genomewide landmarks
for preventive personalized medicine. Front. Biosci. (Schol
Ed.). 2017;9(2):276-306. DOI 10.2741/S488.

Ponomarenko P., Chadaeva I., Rasskazov D., Sharypova E., Kashina
E.V., Drachkova I., Zhechev D., Ponomarenko M., Savinkova L.,
Kolchanov N. Candidate SNP markers of familial and sporadic Alzheimer’s
diseases are predicted by a significant change in the affinity
of TATA-binding protein for human gene promoters. Front.
Aging Neurosci. 2017;9:231. DOI 10.3389/fnagi.2017.00231.

Ponomarenko P., Rasskazov D., Suslov V., Sharypova E., Savinkova L.,
Podkolodnaya O., Podkolodny N., Tverdokhleb N., Chadaeva I.,
Ponomarenko M., Kolchanov N. Candidate SNP markers of chronopathologies
are predicted by a significant change in the affinity of
TATA-binding protein for human gene promoters. BioMed Res. Int.
2016;2016:8642703. DOI 10.1155/2016/8642703.

Ponomarenko P., Savinkova L., Drachkova I., Lysova M., Arshinova T.,
Ponomarenko M., Kolchanov N. A step-by-step model of TBP/TATA
box binding allows predicting human hereditary diseases by single
nucleotide polymorphism. Dokl. Biochem. Biophys. 2008;419:88-
92. DOI 10.1134/S1607672908020117.

Pugh B.F. Purification of the human TATA-binding protein, TBP. Methods
Mol. Biol. 1995;37:359-367. DOI 10.1385/0-89603-288-4:359.

Putlyaeva L.V., Demin D.E., Korneev K.V., Kasyanov A.S., Tatosyan
K.A., Kulakovskiy I.V., Kuprash D.V., Schwartz A.M. Potential
markers of autoimmune diseases, alleles rs115662534(T) and
rs548231435(C), disrupt the binding of transcription factors STAT1
and EBF1 to the regulatory elements of human CD40 gene. Biochemistry
(Mosc). 2018;83(12):1534-1542. DOI 10.1134/S0006297
918120118.

Ramachandrappa S., Kulkarni A., Gandhi H., Ellis C., Hutt R., Roberts
L., Hamid R., Papageorghiou A., Mansour S. SHOX haploinsufficiency
presenting with isolated short long bones in the second
and third trimester. Eur. J. Hum. Genet. 2018;26:350-358. DOI
10.1038/ s41431-017-0080-4.

Rhee H., Pugh B. Genome-wide structure and organization of eukaryotic
pre-initiation complexes. Nature. 2012;483(7389):295-301. DOI
10.1038/nature10799.

Sharypova E., Drachkova I., Kashina E., Rasskazov D., Ponomarenko
P., Ponomarenko M., Kolchanov N., Savinkova L. An experimental
study of the effect of rare polymorphisms of human HBB, HBD
and F9 promoter TATA boxes on the kinetics of interaction with the
TATA-binding protein. Vavilovskii Zhurnal Genetiki i Selektsii =
Vavilov Journal of Genetics and Breeding. 2018;22(1):145-152.
DOI 10.18699/VJ18.342. (in Russian)

Sherry S.T., Ward M.H., Kholodov M., Baker J., Phan L., Smigielski
E.M., Sirotkin K. dbSNP: the NCBI database of genetic variation.
Nucleic Acids Res. 2001;29(1):308-311. DOI 10.1093/nar/29.1.308.

Stahl P., Mielnik A., Barbieri C., Schlegel P., Paduch D. Deletion or underexpression
of the Y-chromosome genes CDY2 and HSFY is associated
with maturation arrest in American men with nonobstructive
azoospermia. Asian J. Androl. 2012;14(5):676-682. DOI 10.1038/
aja.2012.55.

Stajich J., Block D., Boulez K., Brenner S., Chervitz S., Dagdigian C.,
Fuellen G., Gilbert J., Korf I., Lapp H., Lehvaslaiho H., Matsalla
C., Mungall C., Osborne B., Pocock M., Schattner P., Senger M.,
Stein L., Stupka E., Wilkinson M., Birney E. The Bioperl toolkit:
Perl modules for the life sciences. Genome Res. 2002;12(10):1611-
1618. DOI 10.1101/gr.361602.

Telenti A., Pierce L.C., Biggs W.H., di Iulio J., Wong E.H., Fabani
M.M., Kirkness E.F., Moustafa A., Shah N., Xie C., Brewer-ton
S.C., Bulsara N., Garner C., Metzker G., Sandoval E., Perkins
B.A., Och F.J., Turpaz Y., Venter J.C. Deep sequencing of 10,000
human genomes. Proc. Natl. Acad. Sci. USA. 2016;113(42):11901-
11906. DOI 10.1073/pnas.1613365113.

Trovato G.M. Sustainable medical research by effective and comprehensive
medical skills: overcoming the frontiers by predictive,
preventive and personalized medicine. EPMA J. 2014;5(1):14. DOI
10.1186/1878-5085-5-14.

Turnaev I., Rasskazov D., Arkova O., Ponomarenko M., Ponomarenko
P., Savinkova L., Kolchanov N. Hypothetical SNP markers
that significantly affect the affinity of the TATA-binding protein to
VEGFA,
ERBB2, IGF1R, FLT1, KDR, and MET oncogene promoters
as chemotherapy targets. Mol. Biol. (Mosc). 2016;50(1):161-
173. DOI 10.7868/S0026898416010201.

Varzari A., Deyneko I.V., Tudor E., Turcan S. Polymorphisms of glutathione
S-transferase and methylenetetrahydrofolate reductase genes
in Moldavian patients with ulcerative colitis: Genotype-phenotype
correlation. Meta Gene. 2016;7:76-82. DOI 10.1016/j.mgene.2015.
12.002.

Waardenberg A.J., Basset S.D., Bouveret R., Harvey R.P. CompGO:
an R package for comparing and visualizing Gene Ontology enrichment
differences between DNA binding experiments. BMC Bioinformatics.
2015;16:275.

Wu J., Wu M., Li L., Liu Z., Zeng W., Jiang R. dbWGFP: a database
and web server of human whole-genome single nucleotide variants
and their functional predictions. Database (Oxford). 2016;2016:
baw024. DOI 10.1093/database/baw024.

Yoo S., Jin C., Jung D., Choi Y., Choi J., Lee W., Lee S., Lee J., Cha S.,
Kim C., Seok Y., Lee E., Park J. Putative functional variants of
XRCC1 identified by RegulomeDB were not associated with lung
cancer risk in a Korean population. Cancer Genet. 2015;208(1-2):
19-24. DOI 10.1016/j.cancergen.2014.11.004.

Zerbino D.R., Wilder S.P., Johnson N., Juettemann T., Flicek P.R.
The ensembl regulatory build. Genome Biol. 2015;16(1):56. DOI
10.1186/s13059-015-0621-5.

